# Increased expression of Caveolin-1 in both of the vitreous and the proliferating membranes among the patients with proliferative diabetic retinopathy

**DOI:** 10.1038/s41433-022-02292-z

**Published:** 2022-10-26

**Authors:** Hongping Xu, Bo Qin

**Affiliations:** Aier Eye Hospital, Jinan University, No. 2048, Huaqiang South Road, Futian District, Shenzhen, 518032 Guangdong China

**Keywords:** Pharmacology, Pharmaceutics

Proliferative diabetic retinopathy (PDR) is the foremost complication of severe diabetic retinopathy (DR). Caveolin-1, an integral membrane protein, is the principal component of caveolae in membranes and is involved in signal transduction, endocytosis, and molecular regulation [1]. In diabetes mellitus, Caveolin-1 regulates insulin signal transduction and reduces inflammation and oxidative stress in diabetic complications [2]. The recent finding from the mice animal model showed that Caveolin-1 was highly expressed in the proliferative membranes of PDR [3]. Caveolar size and transcytosis were upregulated in the retina of streptozotocin-induced diabetic rats [4]. In addition, Caveolin-1 was also found to be upregulated in rat diabetic retina [5]. Therefore, abnormalities of Caveolin-1 in the retina may be related to the pathogenesis of DR. However, this relationship has not been studied in humans before. Here, Caveolin-1 level both in the vitreous of and in the proliferative membrane of PDR patients were investigated.

The study was approved by the Shenzhen Aier Eye Hospital affiliated to Jinan University review Board and adhered to the tenets of the Declaration of Helsinki. All patients provided signed informed consent before participation in the study. Subjects were 18 years of age or older and had consented to undergo vitrectomy in the flat section. Subjects with a history of psychiatric illness were excluded. A total of 30 individuals were recruited. To obtain vitreous fluid specimens, 18 subjects were recruited, including 11 subjects with PDR as experimental group, and 7 subjects with macular hole but with no history of diabetes as control group. 12 subjects were recruited to obtain clinical specimens of proliferative membranes, including 6 subjects with proliferative diabetic retinopathy as experimental group and 6 subjects with idiopathic macular epiretinal membrane (iERM) but with no history of diabetes as control group. Since the amount of membrane obtained from each patient was not sufficient for Western blotting analysis, we combined three samples for each PDR group and iERM group. After obtaining fresh vitreous fluid, the samples were immediately frozen on dry ice and later analysed by ELISA. Membranes from PDR and iERM patients were obtained and quickly frozen on dry ice for later use for Western blotting analysis. Statistical analysis was performed using SPSS16.0. All data in this study were verified following a normal distribution. Data were shown as means ± SDs. Comparisons were performed using an unpaired t-test to analyse intergroup differences of Caveolin-1 in vitreous fluid samples. Statistical significance was defined as having *P* < 0.05.

ELISA method analysed Caveolin-1 level from the vitreous fluid of 11 patients with PDR and 7 patients with macular hole. Mean normalised Caveolin-1 concentration in PDR vitreous was 12.04 ng/mL (SD 2.31) vs. 7.36 ng/mL (SD 1.36) in macular hole vitreous. There was a statistically significant difference between the two groups (*P* = 0.00186). Western blotting analysis showed that the expression of Caveolin-1 in membranes from PDR groups increased compared with that from iERM groups (fold, PDR1: 1.00, PDR2: 1.49, iERM1: 0.33, iERM2: 0.45). Results are presented in Figs. 1 and 2.

**Fig. 1:**
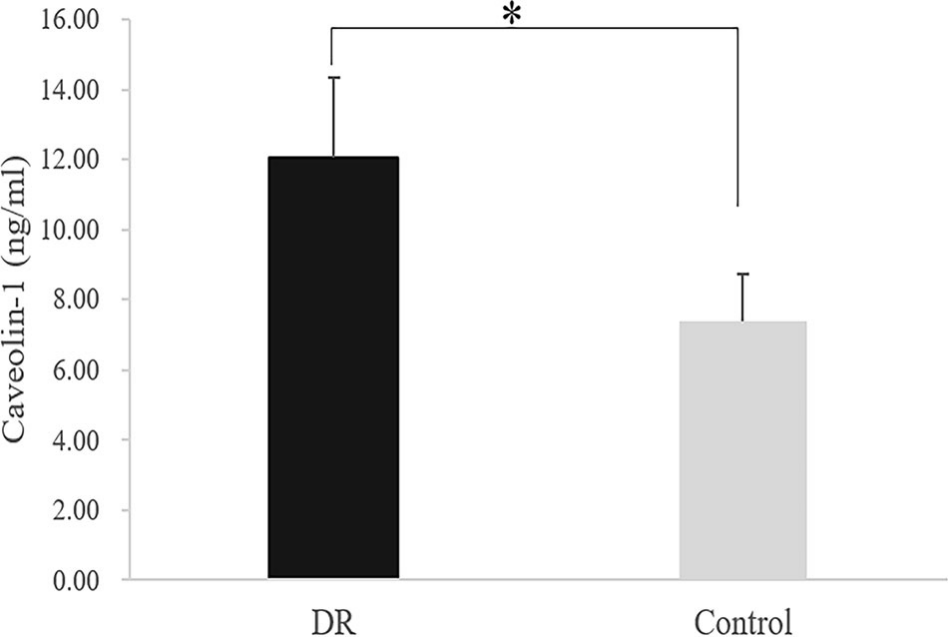
Caveolin-1 level in the vitreous of PDR patients is significantly higher compared to that in the vitreous of macular hole patients. ELISA was used to analyse Caveolin-1 level in the vitreous cavity. PDR group: diabetic retinopathy patients (*n* = 11); control group: macular hole patients (*n* = 7). *P < 0.05.

**Fig. 2:**
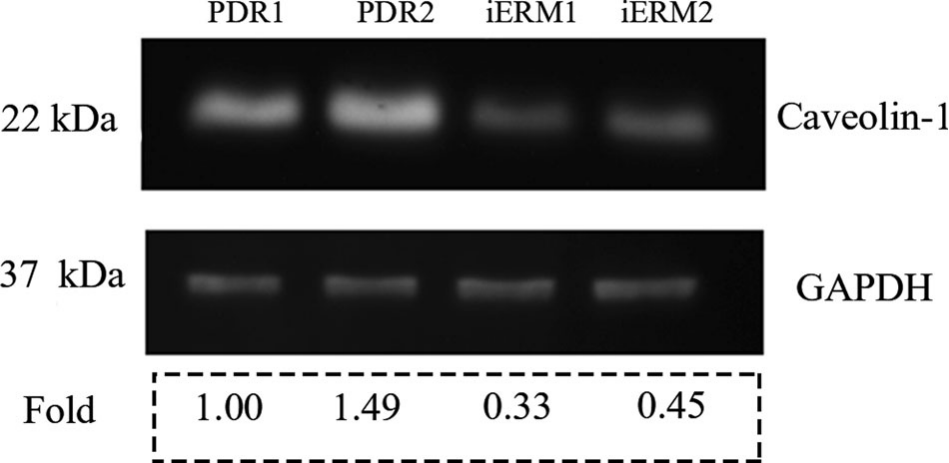
The expression of Caveolin-1 in proliferating membranes of PDR was higher than that of Caveolin-1 in membranes of iERM. Western blotting was used to explore Caveolin-1 expression in membranes. Six cases of proliferating membranes from PDR and six cases of membranes from iERM were divided into four groups: PDR1, PDR2, iERM1, iERM2. For each PDR group, 3 cases of proliferating membranes from PDR were included. For each iERM group, 3 cases of membranes from iERM were included. The Caveolin-1 molecular weight is 22 kDa, and the internal reference molecular weight is 37 kDa. As it could be seen in the picture, Caveolin-1 in proliferating membranes of PDR showed a higher level than that in membranes of iERM. PDR proliferative diabetic retinopathy, *iERM* idiopathic macular epiretinal membrane. GAPDH was the internal reference.

Our study found a significant increase of Caveolin-1 both in the vitreous fluid and in the proliferative membranes from PDR patients. This suggests that Caveolin-1 may be a potential therapeutic target to prevent the development of proliferative membranes in PDR. The Caveolin-1 pathway is a compelling consideration for treatment and further study is necessary to determine optimal conditions for the therapeutic potential.

## Data Availability

Data are available within the article materials.
